# Supplementary material for: Structures of the human LONP1 protease reveal regulatory steps involved in protease activation
Source: Nat Commun. 2021 May 28;12:3239. doi: 10.1038/s41467-021-23495-0 (PMC8163871; doi:10.1038/s41467-021-23495-0)
Supplement: Supplementary file 1 — Supplementary Information [file 41467_2021_23495_MOESM1_ESM.pdf]

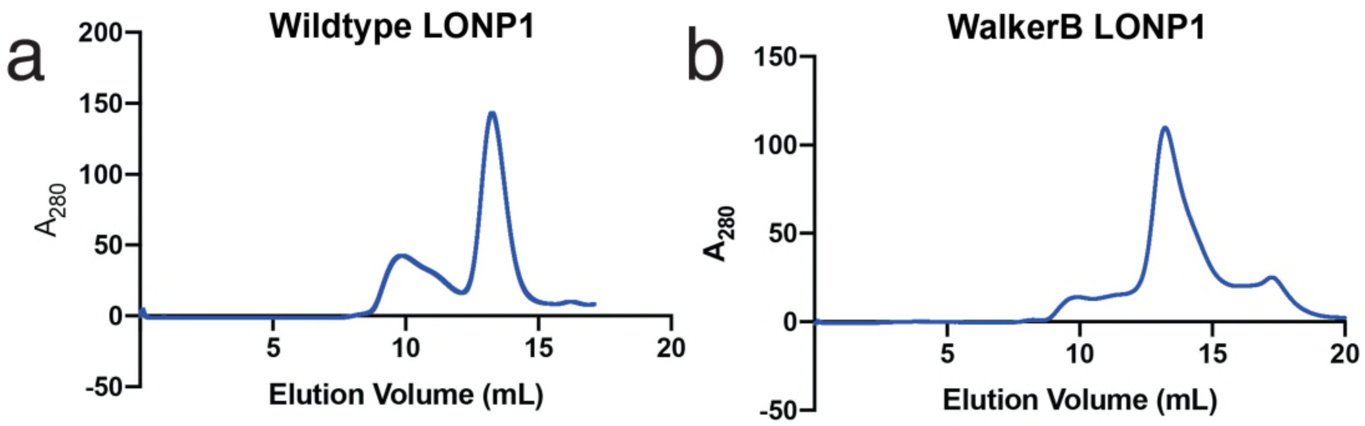

Supplementary Figure 1. Purification of human LONP1 complexes for structural studies. Size-exclusion chromatography (SEC) traces showing elution of recombinantly expressed human LONP1 complexes used for structural analyses eluting around 14 mL for (**A**) wildtype and (**B**) the slowly hydrolyzing E591A (Walker B) mutant LONP1 used for structural studies, indicating a complex size of ~600 kDa.

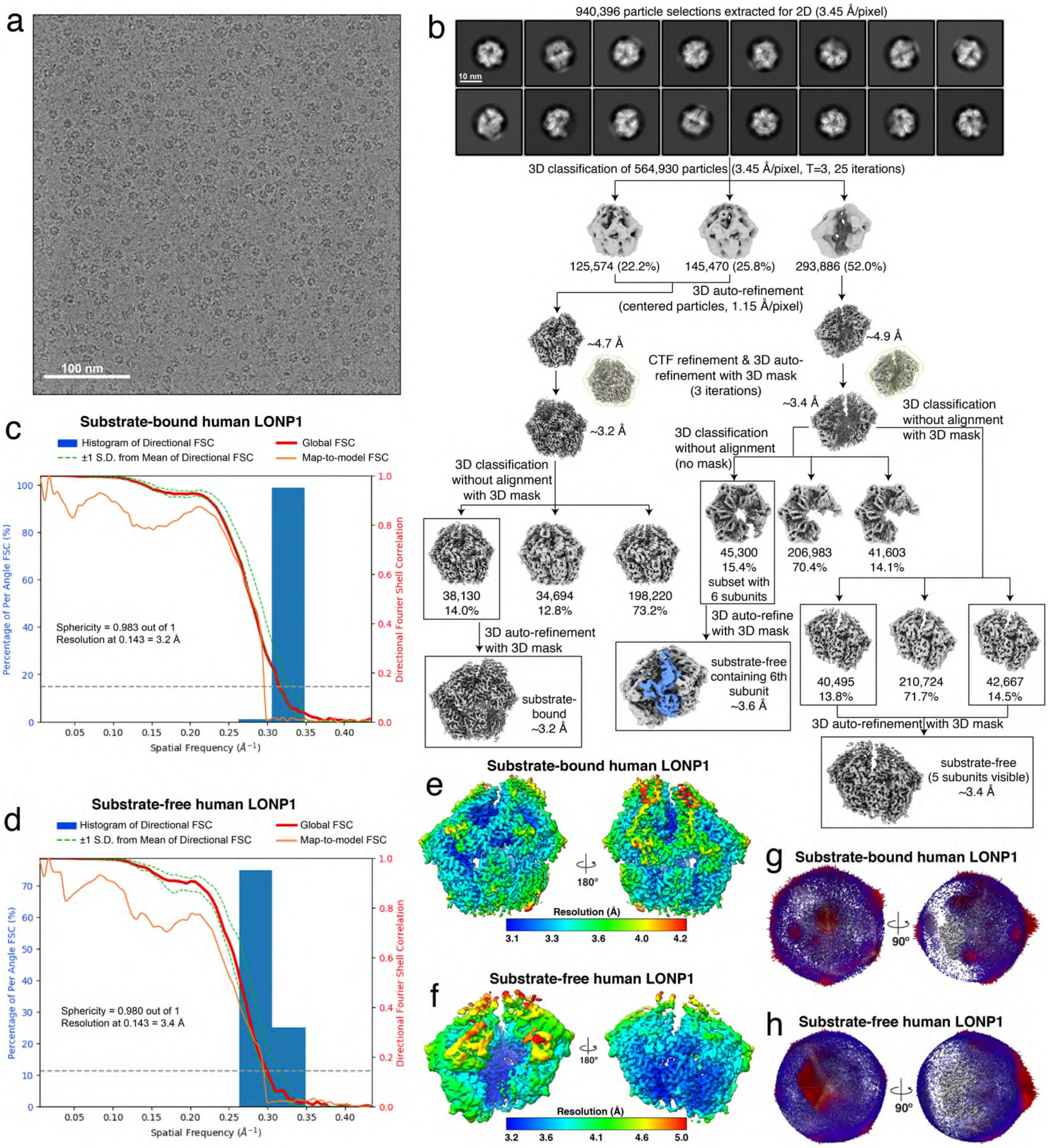

Supplementary Figure 2. Cryo-EM structure determination of the human LONP1 substrate-free and substrate-bound complexes. a. Representative micrograph from cryo-EM data collection. **b.** Cryo-EM data processing scheme followed using RELION 3.1 software[1] to obtain the final 3D reconstructions of substrate-bound and substrate-free human LONP1, including classification revealing a subset of substrate-free particles where the sixth subunit is visible (colored blue). The final maps were used for atomic model building and

refinement. **c-d.** 3-Dimensional Fourier Shell Correlation (3DFSC)[2] of the final reconstruction of substrate-bound (**c**) and substrate-free (**d**) human LONP1 reporting a global resolution of 3.2 Å and 3.4 Å at FSC=0.143, respectively. Both reconstructions showed a high level of sphericity, as calculated by the 3DFSC (0.983 out of 1 for the substrate-bound and 0.980 out of 1 for the substrate-free). **e-f.** Final substrate-bound (**e**) and substrate-free (**f**) reconstructions filtered and colored by local resolution, as calculated using RELION. The final EM densities are resolved to a range of resolutions. The substate-bound complex is mostly resolved to 3.1 Å resolution at the core of the complex, but > 4.0 Å in more flexible regions, such as the 'seam' subunit. In the substrate-free reconstruction, the protease domains of the central subunits are mostly resolved to 3.2 Å resolution, while the peripheral subunits and most of the ATPase domains are resolved to > 4.0 Å. **g-h.** Euler angle distribution plots of the 38,130 and 83,162 particles used in the final reconstruction of the substrate-bound and substrate-free structures, shown from two orthogonal directions.

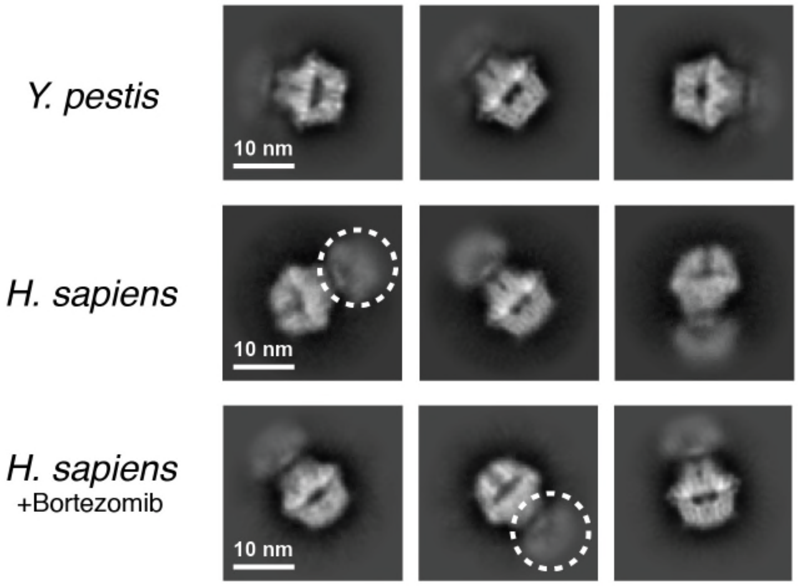

Supplementary Figure 3. 2D averages of *H. sapiens* LONP1 reveal conformationally heterogenous N-terminal domain. The >250 amino acids pertaining to the N-terminal regulatory domain in substrate-bound *Y. pestis* is too flexible to resolve, and observed as a very faint cloud of increased density above the ATPase domains. However, the N-terminal domain in human LONP1, while too flexible to resolve to high resolution, is more readily visible in 2D class averages (highlighted using a hatched circle). All 2D averages were generated with cryoSPARC[3] using identical alignment parameters.

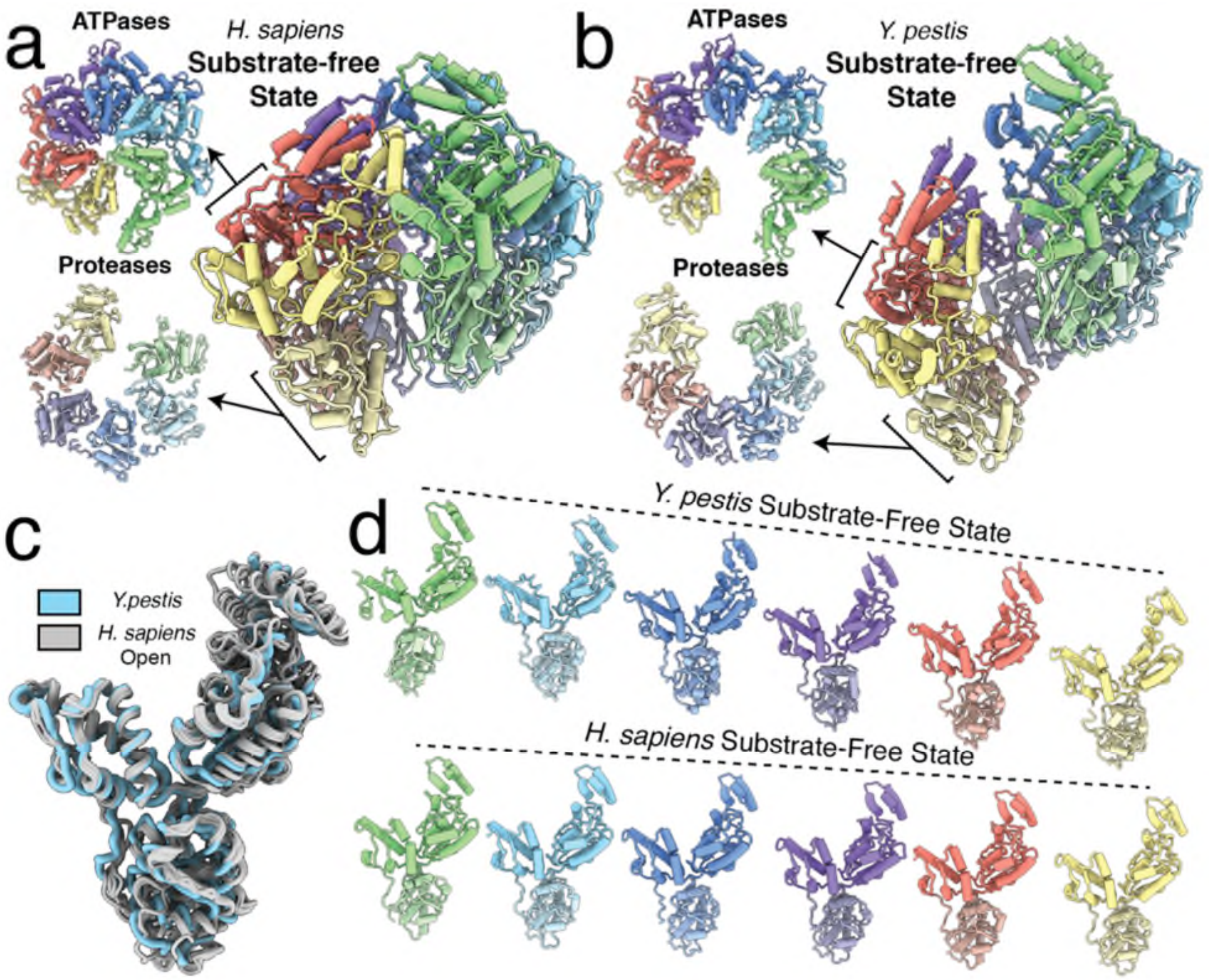

Supplementary Figure 4. Comparison of substrate-free Lon protease structures from *Y. pestis* and *H. sapiens*. a,b. Axial (left) and lateral (right) views of the substrate-free human LONP1 atomic model (**a**) next to the *Y. pestis* Lon substrate-free atomic model[4] (**b**). Axial views of the ATPase and protease domain rings are shown from the exterior of each ring. Subunits are colored as in **Fig. 1**. **c.** Individual subunits from the substrate-free human LONP1 structure (grey) were aligned using a secondary structure-based alignment and subsequently aligned to a subunit from the substrate-free *Y. pestis* structure (light blue). These alignments reveal structural conservation between substrate-free states Lon homologs. **d.** Individual protomers of substrate-free bacterial Lon (top) and substrate-free human LONP1 (bottom) in the intermediate state lined alongside one another relative to the protease domain produced by orienting all the protease domains to a common view. The subunits of the substrate-free *Y. pestis* structure possess a steeper left-handed helical pitch than in the human substrate-free state.

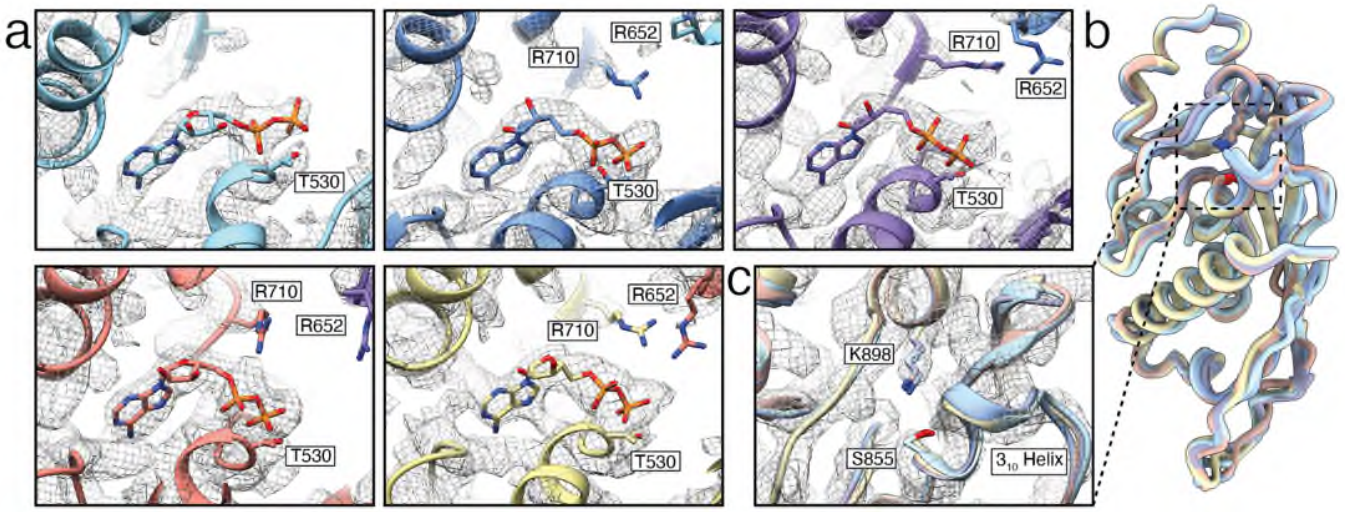

Supplementary Figure 5. Structure of substrate-free LONP1 represents a fully ADP-bound, inactivated conformation. a. The nucleotide binding pockets of all five subunits of substrate-free LONP1 arranged from highest (light blue) to lowest (yellow) subunits, with the cryo-EM density in this region shown as an isosurface mesh contoured at sigma = 3.6. All subunits possess density for nucleotide that is unambiguously consistent with an ADP molecule. **b.** Alignment of all five protease domains of the substrate-free state of LONP1 show that the protease domain adopts a consistently inactivated conformation where a 3_{10} helix blocks entrance of substrates into the proteolytic active site, highlighted by a hatched box. **c.** A zoomed-in view of the proteolytic active sites of all six subunits exhibiting the auto-inhibited conformation of LONP1.

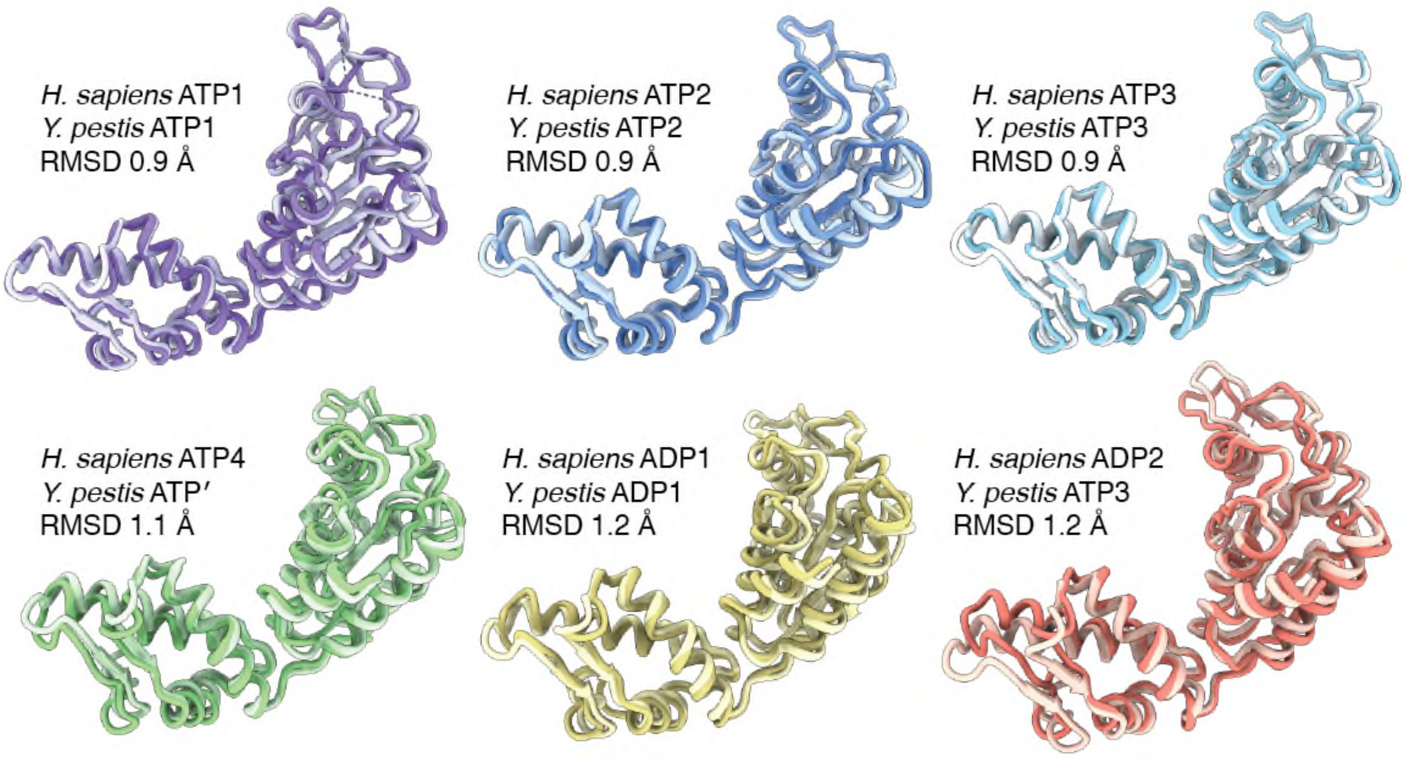

Supplementary Figure 6. ATPase domains of human LONP1 have a nearly identical organization as *Y. pestis* Lon. The AAA+ of the substrate-bound LONP1 conformer are aligned to the corresponding AAA+ domains from substrate-bound *Y. pestis* Lon. All six subunits of the ATPase overlay with RMSDs <1.2 Å.

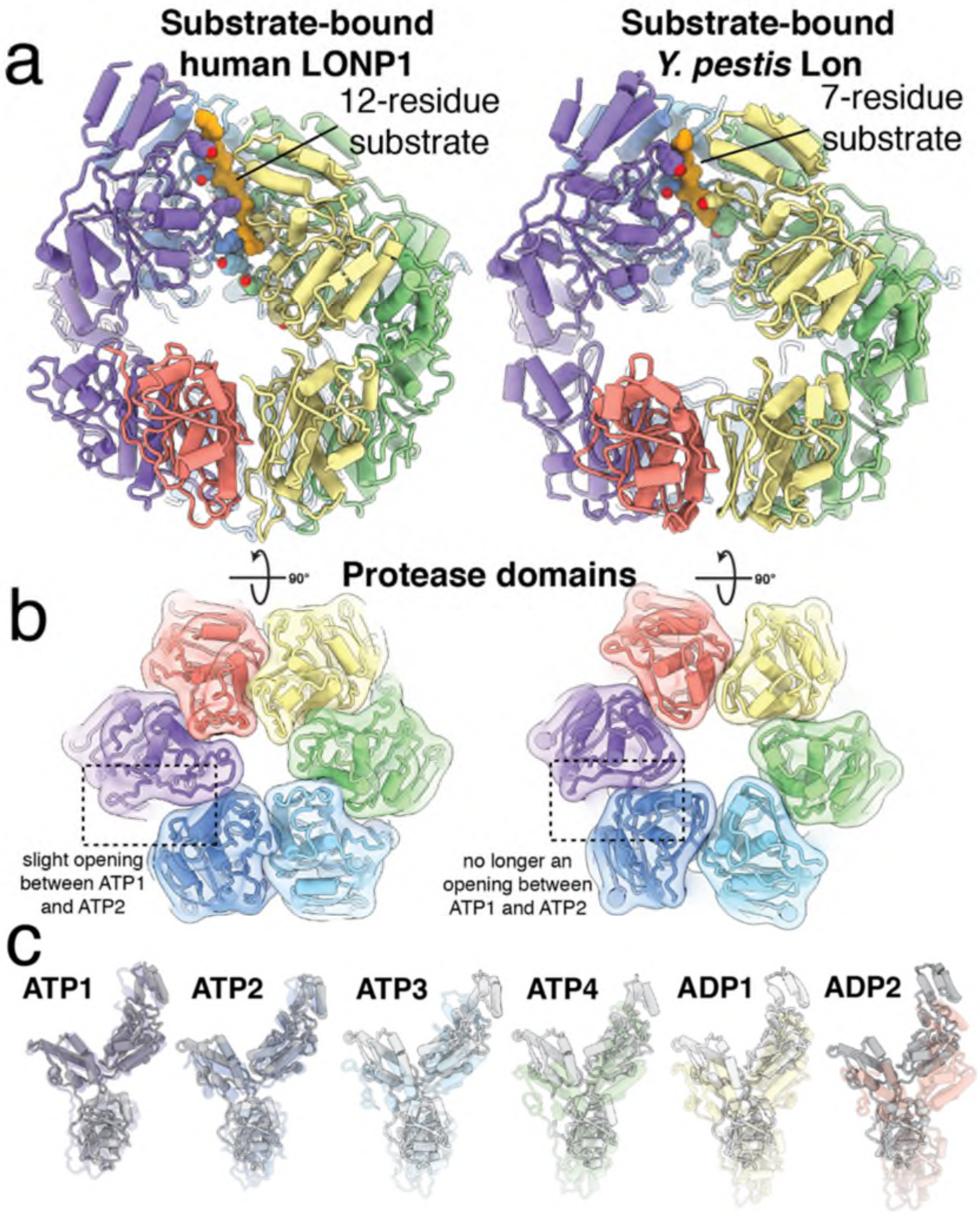

Supplementary Figure 7. Comparison of substrate-bound Lon protease structures from *Y. pestis* and *H. sapiens*. a. Cutaway views of the substrate-bound human LONP1 and substrate-bound *Y. pestis* Lon atomic models[4]. Subunits are colored as in **Fig. 1.** Notably, human LONP1 has a 12-residue substrate trapped in its central channel while *Y. pestis* contains 7 residues, likely due to additional substrate interactions evolved in human LONP1, namely, a tyrosine pore-loop 2. **b.** Axial views of the protease rings emphasize the asymmetry of the substrate-bound LONP1 (left), which has a slight opening between the protease subunits of ATP1 and ATP2, compared to the six-fold symmetric organization of the substrate-bound *Y. pestis* Lon (right).

c. Individual protomers of substrate-bound bacterial Lon aligned side-by-side relative to the protease domain produced by orienting all the proteases to a common view. Subunits from substrate-bound human LONP1 are shown colored as in (**a**) and shown with transparency on top of the subunits of bacterial Lon (colored gray). While the spiraling ATPases sit atop a planar proteolytic ring in bacterial Lon, both AAA+ and protease domains in substrate-bound human LONP1 are asymmetric.

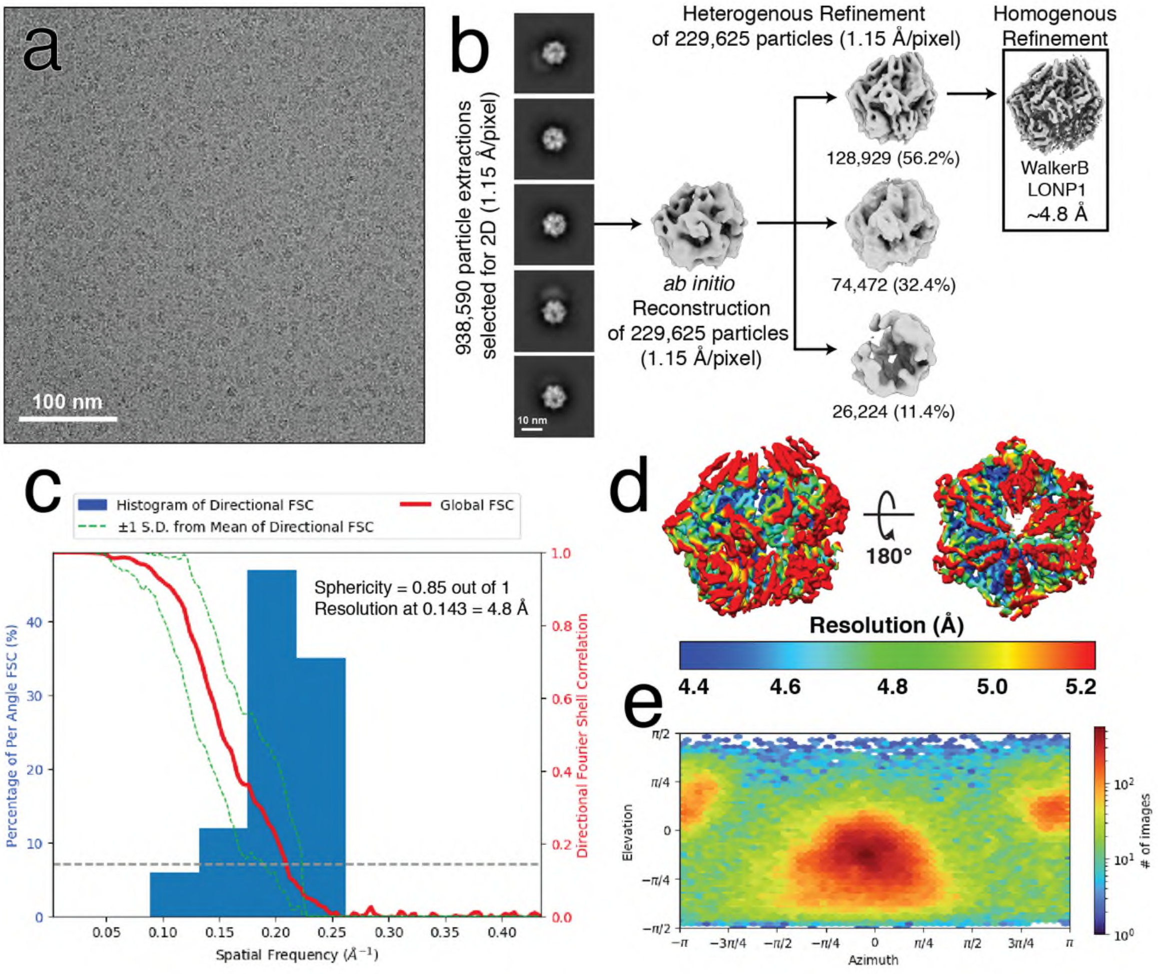

Supplementary Figure 8. Cryo-EM structure determination of the Walker B mutant human LONP1 complex. a. Representative micrograph from cryo-EM data collection. **b.** Cryo-EM data processing scheme using cryoSPARC 3.0.1 software[3,5] to obtain the 3D reconstruction of Walker B mutant human LONP1. **c.** 3D Fourier Shell Correlation (3DFSC)[2] of the final reconstruction of Walker B mutant human LONP1 reporting a global resolution of 4.8 Å at FSC=0.143. **d.** Final Walker B reconstruction filtered and colored by local resolution, as calculated using cryoSPARC, showing that the complex shows a similar range of resolutions as other stabilized LONP1 complexes determined in this study. **e.** Euler angle distribution plot of the 128,929 particles used in the final reconstruction of the Walker B LONP1 structure.

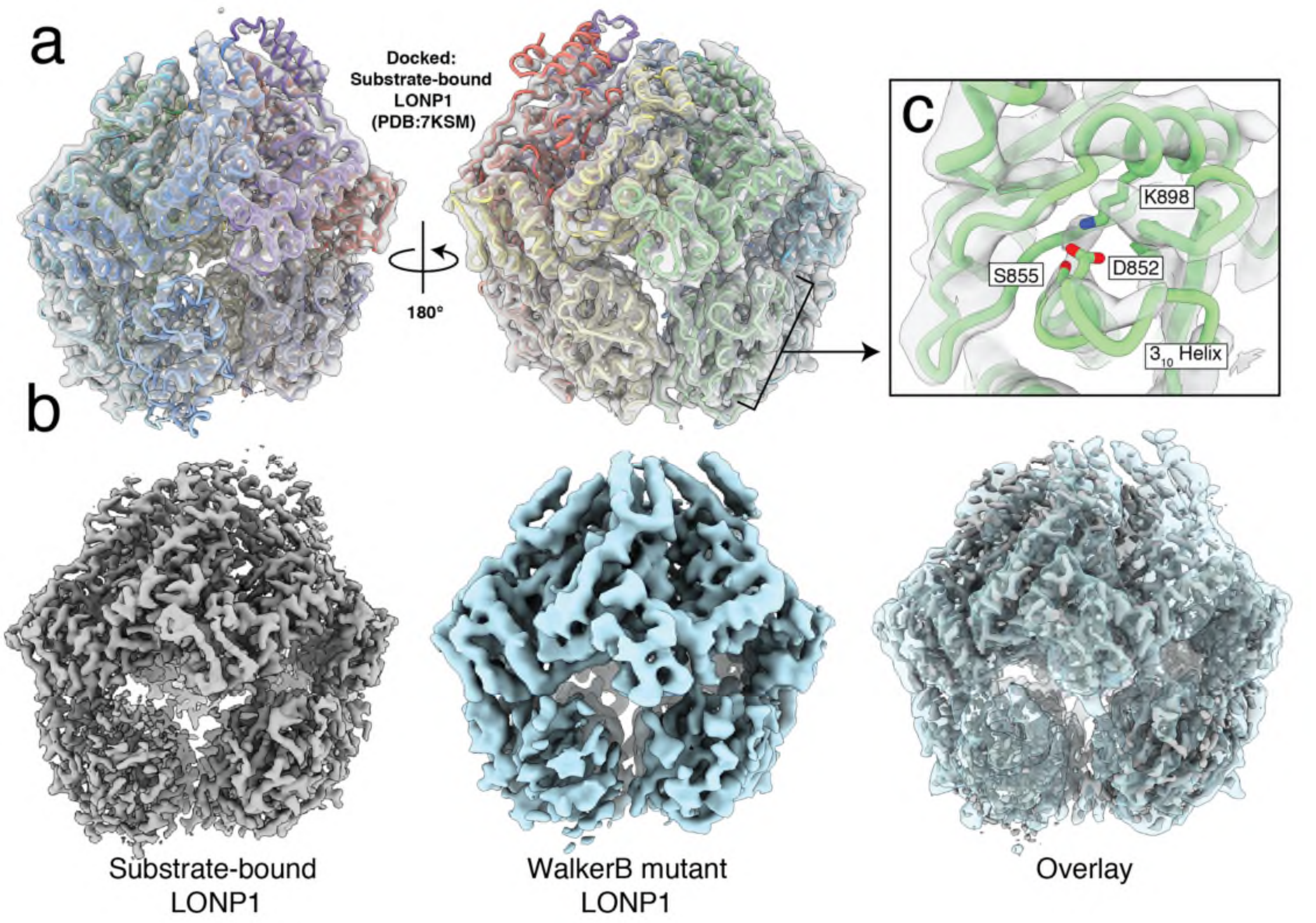

Supplementary Figure 9. Walker B mutant LONP1 resembles substrate-bound LONP1 bound to ATPγS. (**a**) The atomic model of substrate-bound LONP1 (PDB:7KSM) bound to the slowly hydrolyzing ATP analog, ATPγS is shown docked into cryo-EM map of Walker B mutant LONP1. Each of the subunits is colored according to the same coloring scheme as in **Fig. 1c**. The fit of substrate-bound LONP1 into the Walker B cryo-EM map supports our conclusion that the distinct configuration of substrate-bound LONP1 is not dependent upon ATPγS. (**b**) The cryo-EM maps of substrate-bound LONP1 and Walker B mutant LONP1, and an overlay of the two maps, shown side-by-side in the same orientation, showing the similarities of the two structures, including an asymmetric protease ring. (**c**) Zoomed-in view of the protease domain of subunit D in auto-inhibited conformation, where a 3_{10} helix blocks entry of substrates into the proteolytic active site and an inhibitory aspartic acid, D852 prevents formation of the catalytic dyad between S855 and K898.

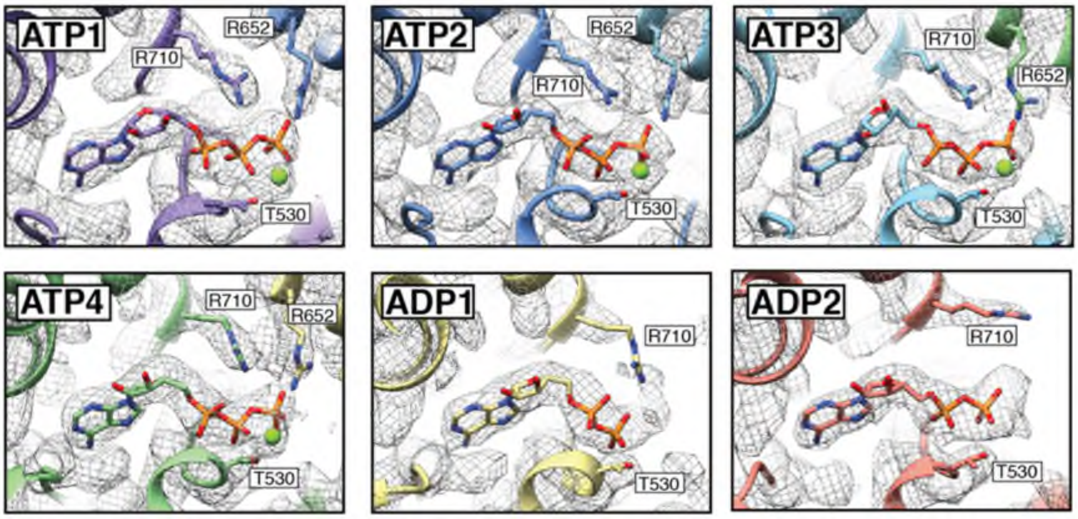

Supplementary Figure 10. Substrate-bound human LONP1 shows nucleotide densities in the nucleotide-binding pocket. The cryo-EM density, shown as an isosurface mesh contoured at sigma=4.0, in the vicinity of the nucleotide binding pocket was of sufficient quality to assign the nucleotide state of each of the subunits. ATP1, ATP2, ATP3, and ATP4 subunits correspond to the ATPγS used to determine this structure coordinated by a magnesium cofactor. The nucleotide density in the ADP1 and ADP2 subunits likely correspond to ADP molecules.

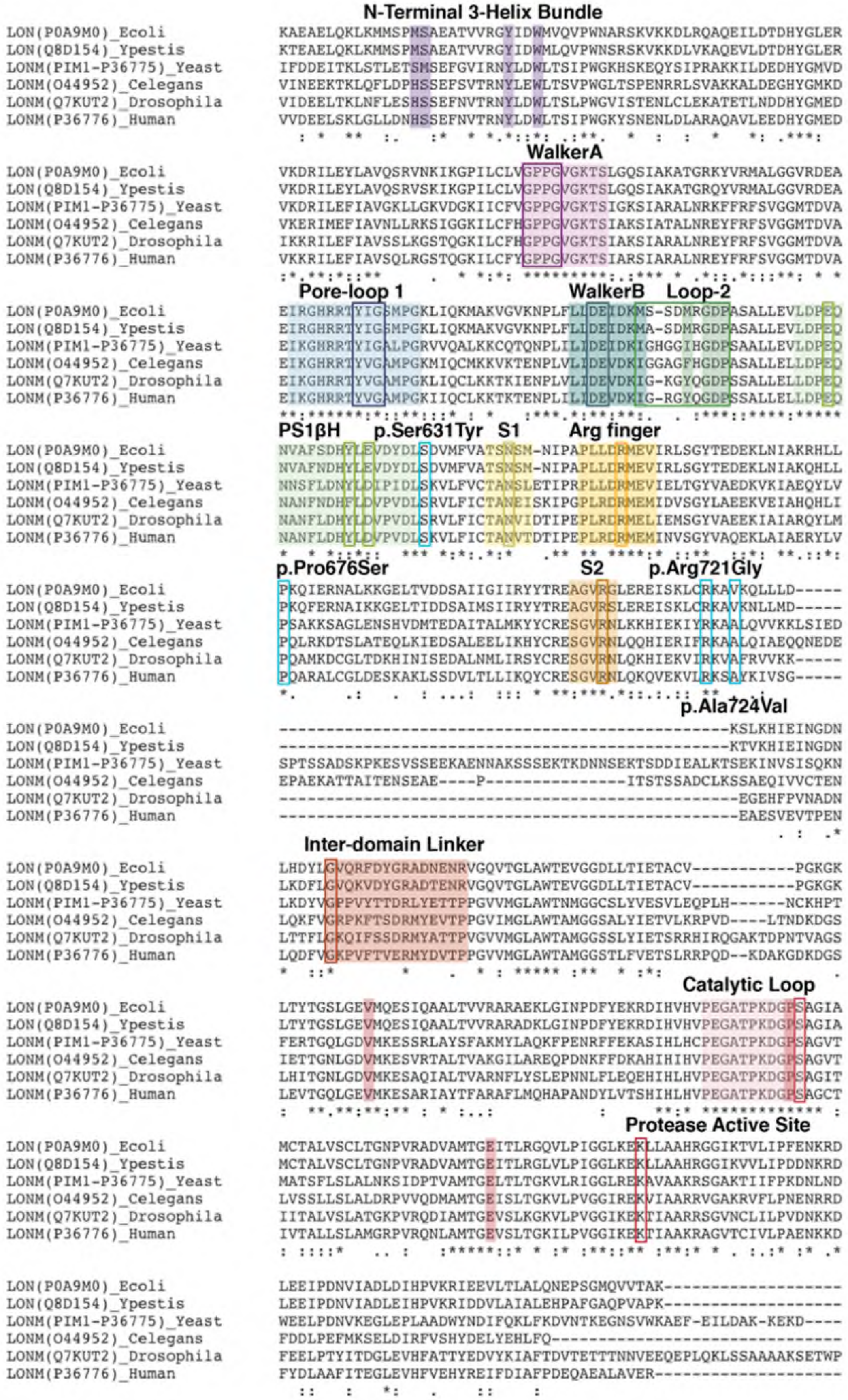

N-Terminal 3-Helix Bundle
LON(P0A9M0)_Ecoli KAEAELQKLKMMSPMSAEATVVRGYIDWMVQVPWNARSKVKKDLRQAQEILDTDHYGLER
LON(Q8D154)_Ypestis KTEAELQKLKMMSPMSAEATVVRGYIDWMLQVPWNSRSKVKKDLVKAQEVLDTDHYGLER
LONM(PIM1-P36775)_Yeast IFDDEITKLSTLETSMSEFGVIRNYLDWLTSIPWGKHSKEQYSIPRAKKILDEDHYGMVD
LONM(O44952)_Celegans VINEEKTKLQFLDPHSSEFSVTRNYLEWLTSVPWGLTSPENRRLSVAKKALDEGHYGMKD
LONM(Q7KUT2)_Drosophila VIDEELTKLNFLESHSSEFNVTRNYLDWLTSLPWGVISTENLCLEKATETLNDDHYGMED
LONM(P36776)_Human VVDEELSKLGLLDNHSSEFNVTRNYLDWLTSIPWGKYSNENLDLARAQAVLEEDHYGMED
WalkerA
LON(P0A9M0)_Ecoli VKDRILEYLAVQSRVNKIKGPILCLVGPPGVGKTSLGQSIAKATGRKYVRMALGGVRDEA
LON(Q8D154)_Ypestis VKDRILEYLAVQSRVSKIKGPILCLVGPPGVGKTSLGQSIAKATGRQYVRMALGGVRDEA
LONM(PIM1-P36775)_Yeast VKDRILEFIAVGKLLGKVDGKIICFVGPPGVGKTSIGKSIARALNRKFFRFSVGGMTDVA
LONM(O44952)_Celegans VKERIMEFIAVNLLRKSIGGKILCFHGPPGVGKTSIAKSIATALNREYFRFSVGGMTDVA
LONM(Q7KUT2)_Drosophila IKKRILEFIAVSSLKGSTQGKILCFHGPPGVGKTSIAKSIARALNREYFRFSVGGMTDVA
LONM(P36776)_Human VKKRILEFIAVSQLRGSTQGKILCFYGPPGVGKTSIARSIARALNREYFRFSVGGMTDVA
Pore-loop 1
WalkerB
Loop-2
LON(P0A9M0)_Ecoli EIRGHRRTYIGSMPGKLIQKMAKVGVKNPLFLLDEIDKMS-SDMRGDPASALLEVLDPEQ
LON(Q8D154)_Ypestis EIRGHRRTYIGSMPGKLIQKMAKVGVKNPLFLLDEIDKMA-SDMRGDPASALLEVLDPEQ
LONM(PIM1-P36775)_Yeast EIKGHRRTYIGALPGRVVQALKKCQTQNPLILIDEIDKIGHGGIHGDPSAALLEVLDPEQ
LONM(O44952)_Celegans EIKGHRRTYVGAMPGKMIQCMKKVKTENPLVLIDEVDKIGGAGFHGDPASALLELLDPEQ
LONM(Q7KUT2)_Drosophila EIKGHRRTYVGAMPGKLIQCLKKTKIENPLVLIDEVDKIG-KGYQGDPSSALLELLDPEQ
LONM(P36776)_Human EIKGHRRTYVGAMPGKIIQCLKKTKTENPLILIDEVDKIG-RGYQGDPSSALLELLDPEQ
PS1βH
p.Ser631Tyr
S1
Arg finger
LON(P0A9M0)_Ecoli NVAFSDHYLEVDYDLSDVMFVATSNSM-NIPAPLLDRMEVIRLSGYTEDEKLNIAKRHLL
LON(Q8D154)_Ypestis NVAFNDHYLEVDYDLSDVMFVATSNSM-NIPAPLLDRMEVIRLSGYTEDEKLNIAKQHLL
LONM(PIM1-P36775)_Yeast NNSFLDNYLDIPIDLSKVLFVCTANSLETIPRPLLDRMEVIELTGYVAEDKVKIAEQYLV
LONM(O44952)_Celegans NANFNDHFLDVPVDLSRVLFICTANEISKIPGPLRDRMEMIDVSGYLAEEKVEIAHQHLI
LONM(Q7KUT2)_Drosophila NANFLDHYLDVPVDLSRVLFICTANVIDTIPEPLRDRMELIEMSGYVAEEKIAIARQYLM
LONM(P36776)_Human NANFLDHYLDVPVDLSKVLFICTANVTDTIPEPLRDRMEMINVSGYVAQEKLAIAERYLV
p.Pro676Ser
S2
p.Arg721Gly
LON(P0A9M0)_Ecoli PKQIERNALKKGELTVDDSAIIGIIRYYTREAGVRGLEREISKLCRKAVKQLLLD-----
LON(Q8D154)_Ypestis PKQFERNAIKKGELTIDDSAIMSIIRYYTREAGVRSLEREISKLCRKAVKNLLMD-----
LONM(PIM1-P36775)_Yeast PSAKKSAGLENSHVDMTEDAITALMKYYCRESGVRNLKKHIEKIYRKAALQVVKKLSIED
LONM(O44952)_Celegans PQLRKDTSLATEQLKIEDSALEELIKHYCRESGVRNLQQHIERIFRKAALQIAEQQNEDE
LONM(Q7KUT2)_Drosophila PQAMKDCGLTDKHINISEDALNMLIRSYCRESGVRNLQKHIEKVIRKVAFRVVKK-----
LONM(P36776)_Human PQARALCGLDESKAKLSSDVLTLLIKQYCRESGVRNLQKQVEKVLRKSAYKIVSG-----
p.Ala724Val
LON(P0A9M0)_Ecoli ---KSLKHIEINGDN
LON(Q8D154)_Ypestis ---KTVKHIEINGDN
LONM(PIM1-P36775)_Yeast SPTSSADSKPKESVSSEEKAENNAKSSSEKTKDNNSEKTSDDIEALKTSEKINVSISQKN
LONM(O44952)_Celegans EPAEKATTAITENSEAE----P---------------ITSTSSADCLKSSAEQIVVCTEN
LONM(Q7KUT2)_Drosophila --EGEHFPVNADN
LONM(P36776)_Human --EAESVEVTPEN
Inter-domain Linker
LON(P0A9M0)_Ecoli LHDYLGVQRFDYGRADNENRVGQVTGLAWTEVGGDLLTIETACV-----------PGKGK
LON(Q8D154)_Ypestis LKDFLGVQKVDYGRADTENRVGQVTGLAWTEVGGDLLTIETACV-----------PGKGK
LONM(PIM1-P36775)_Yeast LKDYVGPPVYTTDRLYETTPPGVVMGLAWTNMGGCSLYVESVLEQPLH------NCKHPT
LONM(O44952)_Celegans LQKFVGRPKFTSDRMYEVTPPGVIMGLAWTAMGGSALYIETVLKRPVD----LTNDKDGS
LONM(Q7KUT2)_Drosophila LTTFLGKQIFSSDRMYATTPVGVVMGLAWTAMGGSSLYIETSRRHIRQGAKTDPNTVAGS
LONM(P36776)_Human LQDFVGKPVFTVERMYDVTPPGVVMGLAWTAMGGSTLFVETSLRRPQD--KDAKGDKDGS
Catalytic Loop
LON(P0A9M0)_Ecoli LTYTGSLGEVMQESIQAALTVVRARAEKLGINPDFYEKRDIHVHVPEGATPKDGPSAGIA
LON(Q8D154)_Ypestis LTYTGSLGEVMQESIQAALTVVRARADKLGINPDFYEKRDIHVHVPEGATPKDGPSAGIA
LONM(PIM1-P36775)_Yeast FERTGQLGDVMKESSRLAYSFAKMYLAQKFPENRFFEKASIHLHCPEGATPKDGPSAGVT
LONM(O44952)_Celegans IETTGNLGDVMKESVRTALTVAKGILAREQPDNKFFDKAHIHIHVPEGATPKDGPSAGVT
LONM(Q7KUT2)_Drosophila LHITGNLGDVMKESAQIALTVARNFLYSLEPNNLFLEQEHIHLHVPEGATPKDGPSAGIT
LONM(P36776)_Human LEVTGQLGEVMKESARIAYTFARAFLMQHAPANDYLVTSHIHLHVPEGATPKDGPSAGCT
Protease Active Site
LON(P0A9M0)_Ecoli MCTALVSCLTGNPVRADVAMTGEITLRGQVLPIGGLKEKLLAAHRGGIKTVLIPFENKRD
LON(Q8D154)_Ypestis MCTALVSCLTGNPVRADVAMTGEITLRGLVLPIGGLKEKLLAAHRGGIKVVLIPDDNKRD
LONM(PIM1-P36775)_Yeast MATSFLSLALNKSIDPTVAMTGELTLTGKVLRIGGLREKAVAAKRSGAKTIIFPKDNLND
LONM(O44952)_Celegans LVSSLLSLALDRPVVQDMAMTGEISLTGKVLPVGGIREKVIAARRVGAKRVFLPNENRRD
LONM(Q7KUT2)_Drosophila IITALVSLATGKPVRQDIAMTGEVSLKGKVLPVGGIKEKTIAARRSGVNCLILPVDNKKD
LONM(P36776)_Human IVTALLSLAMGRPVRQNLAMTGEVSLTGKILPVGGIKEKTIAAKRAGVTCIVLPAENKKD
LON(P0A9M0)_Ecoli LEEIPDNVIADLDIHPVKRIEEVLTLALQNEPSGMQVVTAK------------------
LON(Q8D154)_Ypestis LEEIPDNVIADLEIHPVKRIDDVLAIALEHPAFGAQPVAPK------------------
LONM(PIM1-P36775)_Yeast WEELPDNVKEGLEPLAADWYNDIFQKLFKDVNTKEGNSVWKAEF-EILDAK-KEKD----
LONM(O44952)_Celegans FDDLPEFMKSELDIRFVSHYDELYEHLFQ------------------------------
LONM(Q7KUT2)_Drosophila FEELPTYITDGLEVHFATTYEDVYKIAFTDVTETTTNNVEEQEPLQKLSSAAAAKSETWP
LONM(P36776)_Human FYDLAAFITEGLEVHFVEHYREIFDIAFPDEQAEALAVER-------------------

Supplementary Figure 11. Sequence alignment of Lon protease homologs. ClustalW[6] alignment of the Uniprot sequences of Lon homologs, including cytosolic Lon from *E. coli* and *Y. pestis*, mitochondrial Lon from yeast (Pim1), *C. elegans*, *Drosophila melanogaster*, and humans. This alignment suggests conservation across Lon proteins. Notably, we found several key residues to be strictly conserved in all sequences studied: the N-terminal 3-Helix Bundle (purple boxes around conserved residues studied in *Y. pestis*), P-loop (magenta), pore-loop 1 aromatic-hydrophobic residues (blue), the Walker B motif (teal), Loop-2 (green), pre-sensor 1 beta hairpin insertion (light green), sensor-1 (yellow), a trans-acting arginine finger (orange-yellow box), a cis-acting arginine finger present in sensor-2 (orange), an inter-domain linker containing a glycine residue at its N-terminus (red-orange), and a catalytic loop that is folded into a 3_{10} helix in the proteolytic active site in auto-inhibited Lon and extended in the activated conformation (salmon). Conserved residues involved in stabilizing the activated configuration of the protease domain are highlighted using filled red boxes (V809, P854, and E882). Mutations associated with CODAS syndrome are highlighted using cyan boxes: p.Ser631Tyr, p.Pro676Ser, p.Arg721Gly, and p.Ala724Val. Residues mutated in CODAS syndrome exhibit conservation across Lon homologs, suggesting their importance for Lon protease activity.

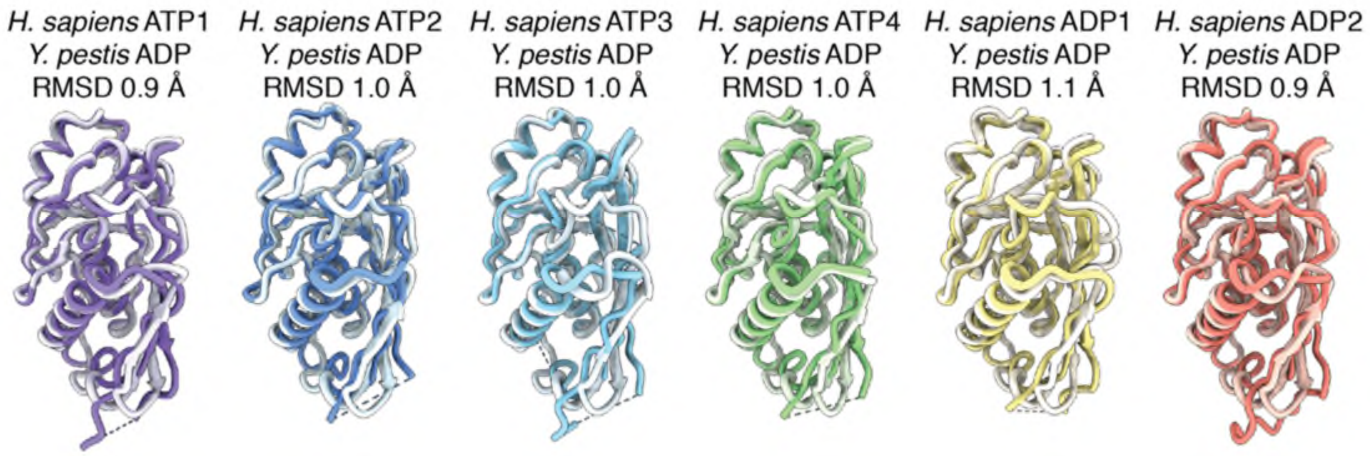

Supplementary Figure 12. Protease domains in the substrate-bound LONP1 structure are auto-inhibited. The protease domains of substrate-bound human LONP1 aligned to their corresponding subunits from the substrate-free *Y. pestis* Lon[4]. All six subunits of the protease domain are in similar conformations, with RMSDs between 0.9-1.1 Å. Despite having substrate trapped in its central channel, the protease domain of semi-closed LONP1 is auto-inhibited by the catalytic loop forming a 3_{10} helix in the active site, similar to substrate-free forms of bacterial and human Lon.

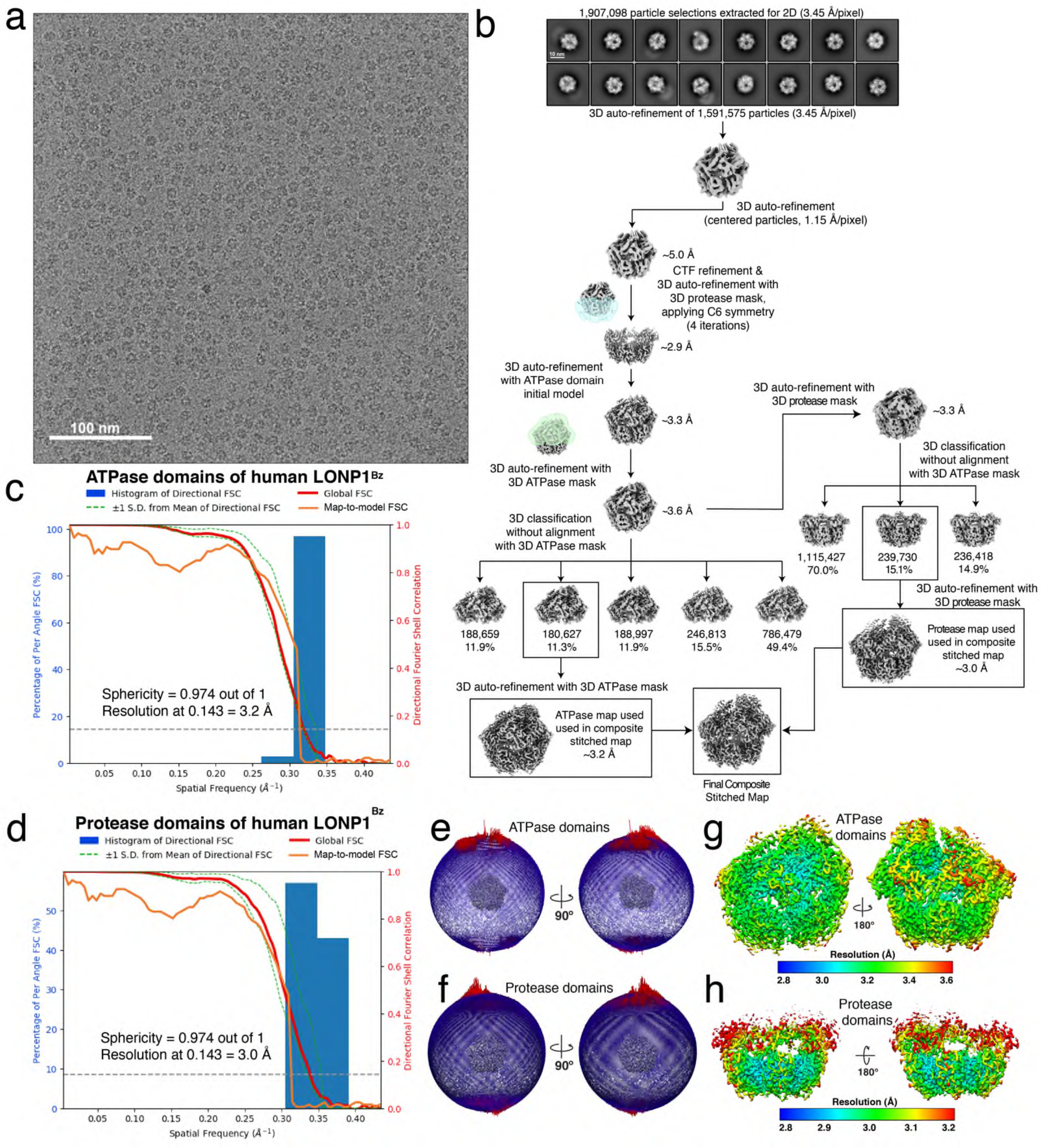

Supplementary Figure 13. Cryo-EM structure determination of the human LONP1 bound to bortezomib. a. Representative micrograph from cryo-EM data collection. **b.** Cryo-EM data processing scheme followed using RELION 3.1 software[1] to obtain the final 3D reconstruction of substrate-bound human LONP1^{Bz}. Final steps included performing focused refinement with a soft mask over the ADP1 and ADP2 subunits to improve resolution of the seam subunits. The focused refinement map was used for atomic model building and refinement. **c-d.**

3DFSCs[2] of the final ATPase (**c**) and protease (**d**) domain reconstructions used in the final composite stitched map of LONP1^{Bz} reporting global resolutions of 3.2 and 3.0 Å at FSC=0.143, respectively and sphericity of 0.974 out of 1 for both. **e-f.** Euler angle distribution plots of the (**e**) 180,627 particles used in the final reconstruction of the ATPase domains and (**f**) 239,730 particles used in the final reconstruction of the protease domains to make the final composite stitched map of LONP1^{Bz} structure. **g-h.** Final ATPase and protease domain reconstructions colored by local resolution calculated using RELION, showing regions resolved to ~3.2 Å at the core of the complex to worse than 3.6 Å in flexible regions for the ATPase domain and ~2.8 Å at the core to worse than 3.2 Å in flexible regions for the protease domain.

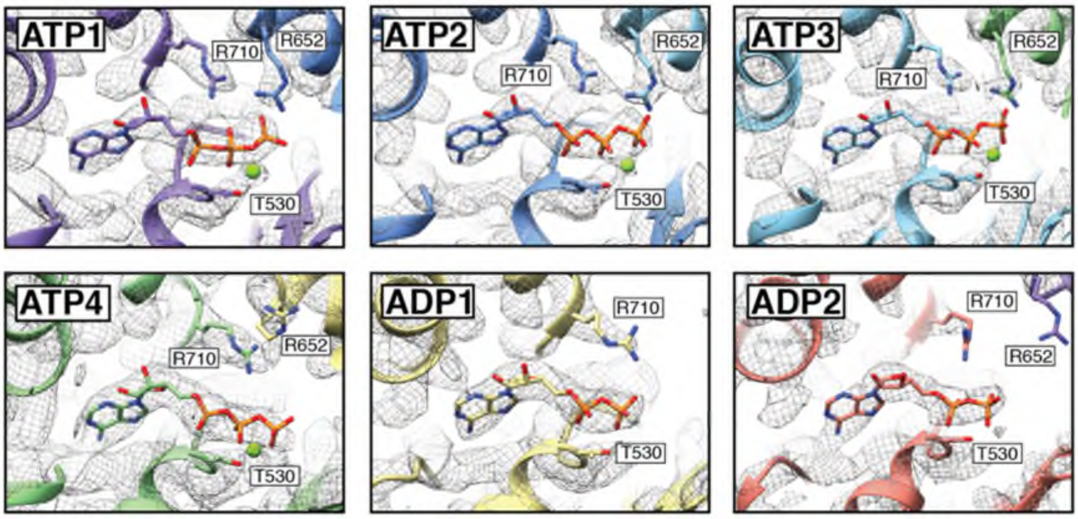

Supplementary Figure 14. LONP1 bound to substrate and bortezomib shows distinct nucleotide densities in the nucleotide-binding pocket. The cryo-EM density, shown as an isosurface mesh contoured at sigma=0.01, in the vicinity of the nucleotide binding pocket was of sufficient quality to unambiguously assign the nucleotide state of each of the subunits. ATP1, ATP2, ATP3, and ATP4 subunits correspond to the ATPγS used to determine this structure coordinated by a magnesium cofactor. The nucleotide density in the ADP1 and ADP2 subunits corresponded to ADP molecules, as there is no apparent gamma density or magnesium.

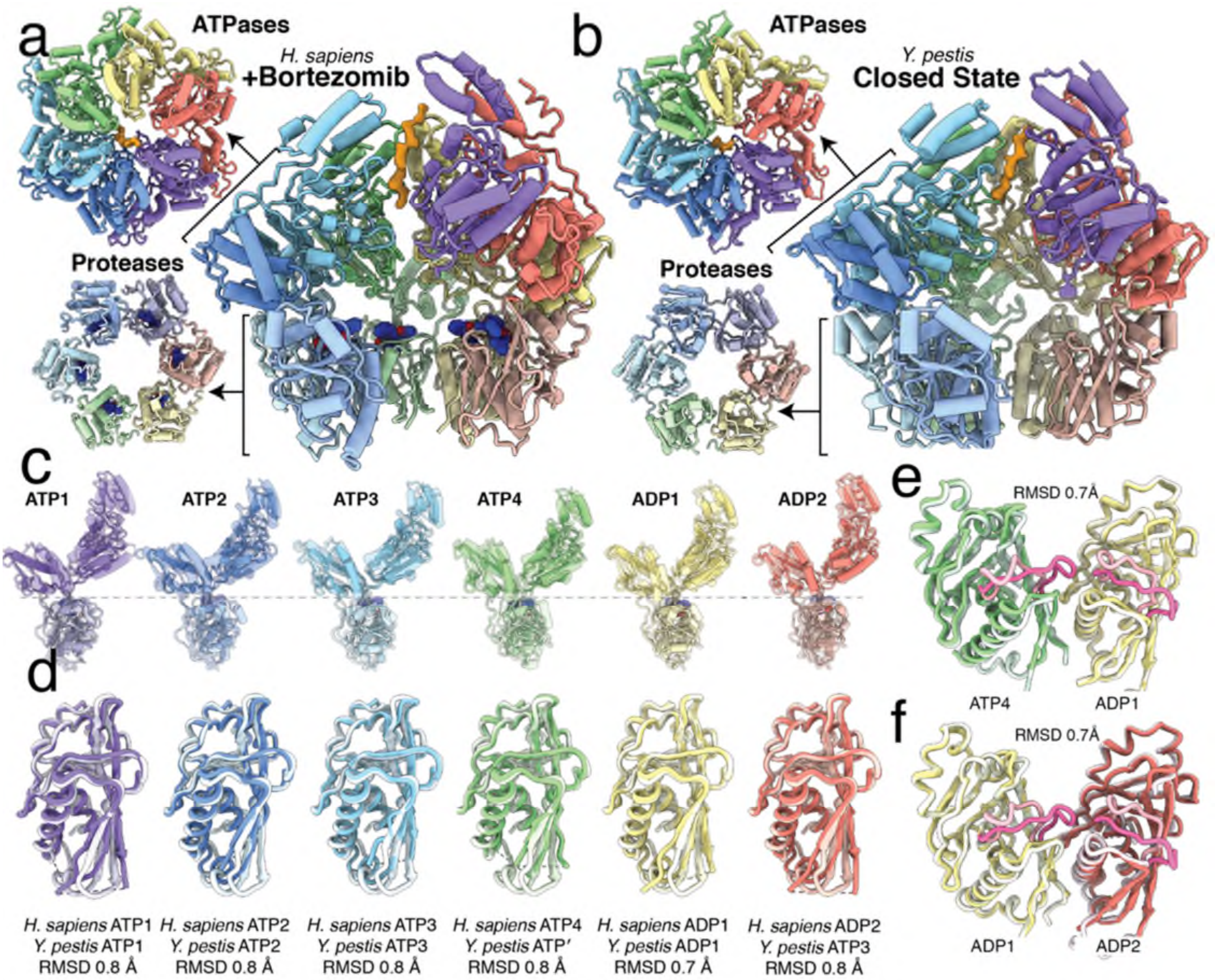

Supplementary Figure 15. Substrate-bound LONP1^{Bz} and substrate-bound *Y. pestis* Lon protease both adopt similar proteolytically active conformations. a,b. Axial and lateral views of the LONP1^{Bz} atomic model (**a**) and the *Y. pestis* substrate-bound state atomic model (**b**).[4] **c.** Individual protomers of LONP1^{Bz} aligned side-by-side relative to the protease domain produced by orienting all the proteases to a common view. The respective subunits from substrate-bound bacterial Lon are shown using a transparent ribbon representation overlaid on the subunits of substrate-bound LONP1^{Bz}. In both cases, the spiraling ATPases sit atop a planar proteolytic ring. **d.** Protease domains from the LONP1^{Bz} conformer are aligned to their corresponding subunits from substrate-bound *Y. pestis* Lon. All six subunits of the protease domain are in similar conformations, with RMSDs between 0.7-0.8 Å. The protease domains of bortezomib-bound LONP1^{Bz} are activated by extending the catalytic loop, allowing catalytic dyad formation and formation of a substrate-binding groove similar to substrate-bound *Y. pestis*

Lon. **e,f**. Secondary structure-based alignments between protease subunit pairs, ATP4 and ADP1 (**e**), and ADP1 and ADP2 (**f**), from substrate-bound LONP1 and $LONP1^{Bz}$ are shown using a ribbon representation. Although the RMSDs for the subunit pair alignment are both 0.7 A, the catalytic serine-containing loop (highlighted in pink) is divergent between the substrate-bound LONP1 and $LONP1^{Bz}$ due to the proteases adopting auto-inhibited (light pink) and activated (dark pink) conformations, respectively.

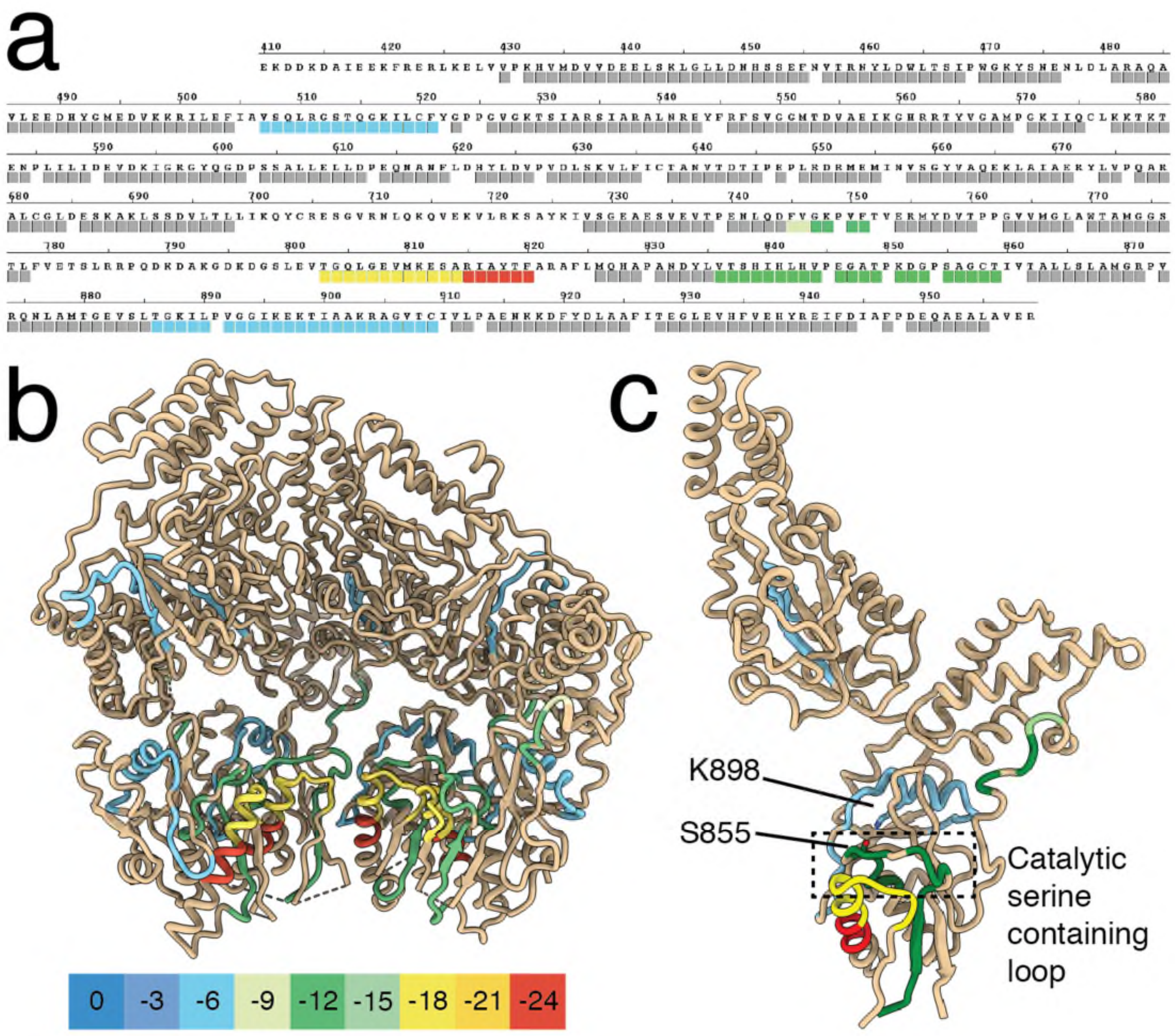

Supplementary Figure 16. Hydrogen-deuterium exchange mass spectrometry shows a conformational change in the protease domains upon binding bortezomib. a. Amino acid sequence of human mitochondrial Lon. Colored boxes located underneath the amino acid sequence denote digested peptides detected in mass spectrometry experiments. Regions with no change in exchange upon the addition of Bortezomib have grey boxes while regions with reduced D_2O exchange upon the addition of Bortezomib are colored from blue to red based on the scale shown in panel **b**; D_2O exchange decreased by 0-6% in regions colored blue while regions colored in red depict where the greatest reduction in D_2O exchange occurred, by ~24%. Regions with most D_2O exchange included residues 803-814 and 815-820 colored yellow and red, respectively, belong to an alpha helix and flexible loop framing the catalytic loop the activated conformation of the protease active site. These regions

likely experience a reduction in D_2O exchange due to protease symmetrization and ring closure upon the addition of Bortezomib. **b.** Peptides that were detected as having a change in D2O exchange rate during hydrogen-deuterium exchange mass spectrometry are colored using the shown scale. **c.** An isolated subunit from (**b**) with a hatched box highlighting the catalytic serine-containing loop and labels for catalytic residues S855 and K898 showing reduction in D_2O exchange in proximity of the proteolytic active site in the presence of Bortezomib.

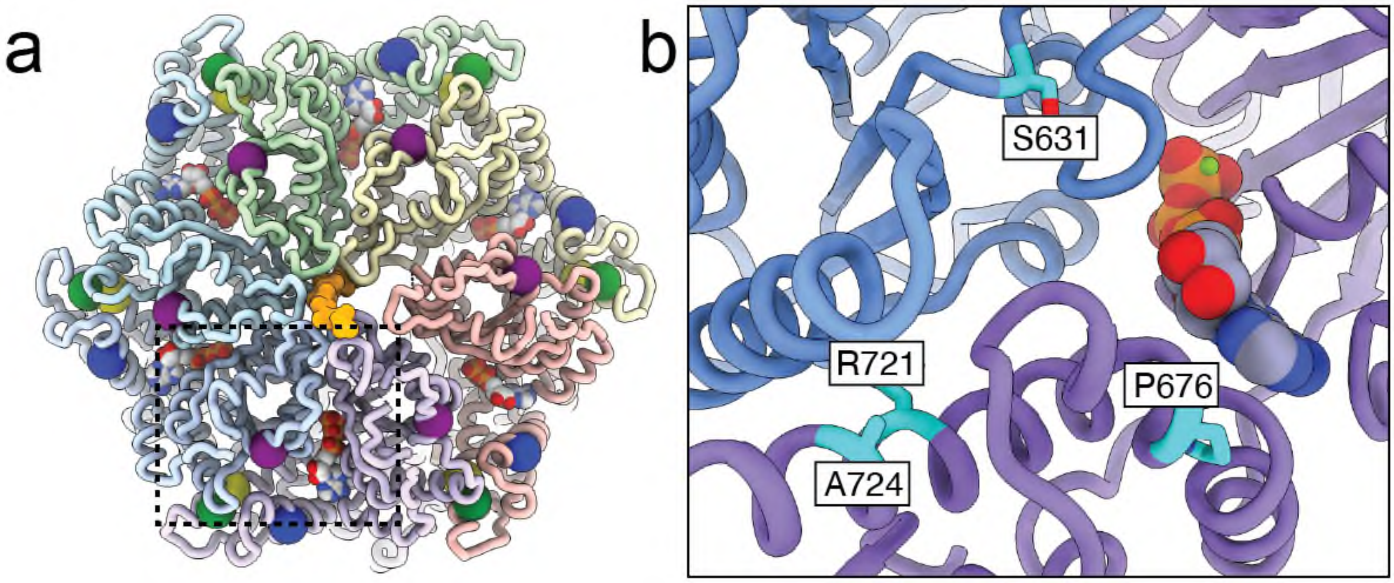

Supplementary Figure 17. Mutations associated with CODAS syndrome localize to inter-subunit interfaces. a. The locations of the following point mutations associated with CODAS syndrome are denoted on the structure of the substrate-bound human LONP1 with spheres: p.Ser631Tyr (purple), p.Pro676Ser (dark blue), p.Arg721Gly (light blue), and p.Ala724Val (green). **b.** A close-up of the nucleotide binding pocket formed between ATP1 (purple) and ATP2 (blue) subunits. Residues bearing mutations associated with CODAS Syndrome (S631, P676, R721, and A724) are located at the inter-subunit interfaces, potentially playing a role in nucleotide binding, hydrolysis, or associated allostery. These mutations likely perturb inter-subunit interactions required for ATP-driven substrate translocation.

Supplementary Table 1. CryoEM data collection, refinement, and validation statistics

	**Substrate-free LONP1 EMDB: 23019 PDB: 7KSL**	**Substrate-bound LONP1 EMDB: 23020 PDB: 7KSM**	**Walker B LONP1 EMDB: 23320**	**Substrate-bound LONP1^{Bz} EMDB: 23013 PDB: 7KRZ**
**Data collection**				
Microscope	Talos Arctica		Talos Arctica	Talos Arctica
Voltage (keV)	200		200	200
Detector	K2 Summit		K2 Summit	K2 Summit
Magnification (nominal/calibrated)	36,000X / 43,478X		36,000X / 43,478X	36,000X / 43,478X
Exposure navigation	Image shift to 16 holes		Image shift to 4 holes	Image shift to 16 holes
Data acquisition software	Leginon[7]		Leginon	Leginon
Total electron exposure ($e^-/Å^2$)	50		50	50
Exposure rate (e^-/pixel/sec)	5.8		5.6	6.3
Frame length (ms)	100		200	200
Number of frames per micrograph	114		59	52
Pixel size (Å)	1.15		1.15	1.15
Defocus range (µm)	-0.8 to -1.5		-0.8 to -1.5	-0.8 to -1.5
Micrographs collected (no.)	2,912		2,415	4,774
**Reconstruction**				
Image processing package	Relion		CryoSparc	Relion
Total extracted particles (no.)	940,396		938,590	2,736,565
Refined particles (no.)	564,930		229,625	1,539,141
Final particles (no.)	83,162	38,130	128,929	532,298
Symmetry imposed	C1	C1	C1	C1
Resolution (Å)				
FSC 0.5 (unmasked / masked)	4.4 / 3.8	4.2 / 3.6	8.3 / 6.7	ATPase: 4.4 / 3.6<br>Protease: 3.1 / 2.9
FSC 0.143 (unmasked / masked)	3.8 / 3.4	3.6 / 3.2	6.9 / 4.8	ATPase: 3.7 / 3.2<br>Protease: 3.0 / 2.7
Resolution range (local)	3.2 – 5.0	3.1 – 4.2	4.4 – 5.2	ATPase: 2.8 – 3.6<br>Protease: 2.8 – 3.2
3DFSC Sphericity	0.980 out of 1	0.983 out of 1	0.85 out of 1	0.986 out of 1
Accuracy of translations / rotations	0.82 / 0.45	0.65 / 0.35	N/A	0.82 / 1.7
Sharpening B-factor ($Å^2$)	-30.1	-7.1	-186.4	-157.2
**Model Composition**				
Protein residues	2,551	3,137		3,127
Ligands	5	22		28
**Model Refinement**				
Refinement package	Phenix[8]	Phenix		Phenix
CC (volume / mask)	0.80 / 0.81	0.85 / 0.85		0.86 / 0.87
R.m.s. deviations				
Bond lengths	0.01	0.01		0.01
Bond angles (°)	0.77	0.72		0.67
**Validation**				
Map-to-model FSC 0.5	4.00	3.61		3.2
Ramachandran (%)				
Outliers	0.00	0.00		0.00
Allowed	8.67	4.44		5.83
Favored	91.33	95.56		4.17
MolProbity score[9]	1.98	1.74		1.81
Poor rotamers (%)	0.12	0.08		0.19
Clashscore (all atoms)	8.76	7.82		7.63
C-beta deviations	0.00	0.00		0.00
CaBLAM Outliers (%)[9988]	4.15	2.37		3.03
EMRinger Score[10]	2.24	3.00		3.45

Supplementary Table 2. Primers used in this study.

Primer Name	Purpose	Sequence
hLON_QC_E884A_REV	QuikChange	caggcaggatcttgcccgtgagggagacTGCgccagtcatggccagattctgccggac
hLON_QC_E884A_FWD	QuikChange	gtccggcagaatctggccatgactggcGCAgtctccctcacgggcaagatcctgcctg
hLON_QC_P854A_REV	QuikChange	gtgacgatggtgcagcctgcgctTGCgccgtccttgggggtggcgccctc
hLON_QC_P854A_FWD	QuikChange	gagggcgccacccccaaggacggcGCAagcgcaggctgcaccatcgtcac
hLON_QC_V809A_REV	QuikChange	ctatgcgggcgctctccttcatCGCctcccccagctggcctgtcac
hLON_QC_V809A_FWD	QuikChange	gtgacaggccagctgggggagGCGatgaaggagagcgcccgcatag
hLON_QC_Pore2(YtoA)_REV	QuikChange	cagtgccgacgacgggtcccctgGGCgcctcggccgatcttgtccacctc
hLON_QC_Pore2(YtoA)_FWD	QuikChange	gaggtggacaagatcggccgaggcGCCcaggggacccgtcgtcggcactg
hLon_QC_ProteaseInactive(StoA)_FWD	QuikChange	gagggcgccacccccaaggacggcccaGCCgcaggctgcaccatcgtcacggccctg
hLon_QC_ProteaseInactive(StoA)_REV	QuikChange	cagggccgtgacgatggtgcagcctgcGGCtgggccgtccttgggggtggcgccctc
hLon_QC_WalkerB(EtoA)_FWD	QuikChange	gagaacccctgatcctcatcgacgCggtggacaagatcggccgaggctac
hLon_QC_WalkerB(EtoA)_REV	QuikChange	gtagcctcggccgatcttgtccaccGcgtcgatgaggatcaggggttctc
hLon_QC_Pore1(YtoA)_FWD	QuikChange	gagatcaagggccacaggcggaccGCCgtgggcgccatgcccgggaagatc
hLon_QC_Pore1(YtoA)_REV	QuikChange	gatcttcccgggcatggcgcccacGGCggtccgcctgtggcccttgatctc
hLon_pET20b_NtermHisTag_RV+	Gibson Assembly	CAGGTTttcgtgatgatggtgatgatgcatatgtatatctccttcttaaag
hLon_pET20b_NtermHisTag_FI+	Gibson Assembly	catcacgaaAACCTGTATTTTCAGGGAgcacacatgatgacgatccccgatgtg
hLon_pET20b_NtermHisTag_RI+	Gibson Assembly	gttagcagccggatctcaccgttccacggccagcgcctc
hLon_pET20b_NtermHisTag_FV-	Gibson Assembly	tgagatccggctgctaac

SUPPLEMENTARY REFERENCES

1 Zivanov, J. *et al.* New tools for automated high-resolution cryo-EM structure determination in RELION-3. *Elife* **7**, doi:10.7554/eLife.42166 (2018).

2 Tan, Y. Z. *et al.* Addressing preferred specimen orientation in single-particle cryo-EM through tilting. *Nat Methods* **14**, 793-796, doi:10.1038/nmeth.4347 (2017).

3 Punjani, A., Rubinstein, J. L., Fleet, D. J. & Brubaker, M. A. cryoSPARC: algorithms for rapid unsupervised cryo-EM structure determination. *Nat Methods* **14**, 290-296, doi:10.1038/nmeth.4169 (2017).

4 Shin, M. *et al.* Structural basis for distinct operational modes and protease activation in AAA+ protease Lon. *Sci Adv* **6**, eaba8404, doi:10.1126/sciadv.aba8404 (2020).

5 Punjani, A., Zhang, H. & Fleet, D. J. Non-uniform refinement: adaptive regularization improves single-particle cryo-EM reconstruction. *Nat Methods* **17**, 1214-1221, doi:10.1038/s41592-020-00990-8 (2020).

6 Thompson, J. D., Higgins, D. G. & Gibson, T. J. CLUSTAL W: improving the sensitivity of progressive multiple sequence alignment through sequence weighting, position-specific gap penalties and weight matrix choice. *Nucleic Acids Res* **22**, 4673-4680, doi:10.1093/nar/22.22.4673 (1994).

7 Suloway, C. *et al.* Automated molecular microscopy: the new Leginon system. *J Struct Biol* **151**, 41-60, doi:10.1016/j.jsb.2005.03.010 (2005).

8 Adams, P. D. *et al.* PHENIX: a comprehensive Python-based system for macromolecular structure solution. *Acta Crystallogr D Biol Crystallogr* **66**, 213-221, doi:10.1107/S0907444909052925 (2010).

9 Prisant, M. G., Williams, C. J., Chen, V. B., Richardson, J. S. & Richardson, D. C. New Tools in MolProbity Validation: CaBLAM for CryoEM Backbone, UnDowser to Rethink "Waters", and NGL Viewer to Recapture Online 3D Graphics. *Protein Sci*, doi:10.1002/pro.3786 (2019).

10 Barad, B. A. *et al.* EMRinger: side chain-directed model and map validation for 3D cryo-electron microscopy. *Nat Methods* **12**, 943-946, doi:10.1038/nmeth.3541 (2015).
